# The cause-and-effect relationship between gut microbiota abundance and carcinoid syndrome: a bidirectional Mendelian randomization study

**DOI:** 10.3389/fmicb.2023.1291699

**Published:** 2023-12-22

**Authors:** Zexin Zhang, Dongting Li, Fengxi Xie, Gulizeba Muhetaer, Haibo Zhang

**Affiliations:** ^1^The Second Clinical College of Guangzhou University of Chinese Medicine, Guangzhou, China; ^2^The Affiliated Guangzhou Hospital of TCM of Guangzhou University of Chinese Medicine, Guangzhou, China; ^3^Maoming Hospital, Guangzhou University of Chinese Medicine, Guangzhou, China; ^4^The Second Affiliated Hospital of Guangzhou University of Chinese Medicine, Guangzhou, China; ^5^Guangdong Key Laboratory of Clinical Research of Chinese Medicine, Guangzhou, China; ^6^Guangdong Joint Laboratory of Guangdong, Hong Kong and Macao Chinese Medicine and Immune Diseases, Guangzhou, China; ^7^State Key Laboratory of Wet Certificate of Chinese Medicine Jointly Built by the Province and the Ministry, Guangzhou, China

**Keywords:** cause-and-effect relationship, gut microbiota abundance, carcinoid syndrome, active substances, Mendelian randomization study

## Abstract

**Objective:**

Carcinoid syndrome (CS) commonly results from neuroendocrine tumors. While active substances are recognized as the main causes of the typical symptoms such as diarrhea and skin flush, the cause-and-effect relationship between gut microbiota abundance and CS remains unclear.

**Methods:**

The Single Nucleotide Polymorphisms (SNPs) related to gut microbiota abundance and CS were obtained from the GWAS summary data. The inverse variance weighted (IVW) method was used to assess the causal relationship between gut microbiota abundance and CS. Additionally, the MR-Egger, Weighted Median model, and Weighted model were employed as supplementary approaches. The heterogeneity function of the TwoSampleMR package was utilized to assess whether SNPs exhibit heterogeneity. The Egger intercept and Presso test were used to assess whether SNPs exhibit pleiotropy. The Leave-One-Out test was employed to evaluate the sensitivity of SNPs. The Steiger test was utilized to examine whether SNPs have a reverse causal relationship. A bidirectional mendelian randomization (MR) study was conducted to elucidate the inferred cause-and-effect relationship between gut microbiota abundance and CS.

**Results:**

The IVW results indicated a causal relationship between 6 gut microbiota taxa and CS. Among the 6 gut microbiota taxa, the genus Anaerofilum (IVW OR: 0.3606, 95%CI: 0.1554–0.8367, *p*-value: 0.0175) exhibited a protective effect against CS. On the other hand, the family Coriobacteriaceae (IVW OR: 3.4572, 95%CI: 1.0571–11.3066, *p*-value: 0.0402), the genus Enterorhabdus (IVW OR: 4.2496, 95%CI: 1.3314–13.5640, *p*-value: 0.0146), the genus Ruminiclostridium6 (IVW OR: 4.0116, 95%CI: 1.2711–12.6604, *p*-value: 0.0178), the genus Veillonella (IVW OR: 3.7023, 95%CI: 1.0155–13.4980, *p*-value: 0.0473) and genus Holdemanella (IVW OR: 2.2400, 95%CI: 1.0376–4.8358, *p*-value: 0.0400) demonstrated a detrimental effect on CS. The CS was not found to have a reverse causal relationship with the above 6 gut microbiota taxa.

**Conclusion:**

Six microbiota taxa were found to have a causal relationship with CS, and further randomized controlled trials are needed for verification.

## Background

Carcinoid syndrome (CS) refers to a series of symptoms mediated by various biologically active substances secreted by neuroendocrine tumors (NETs), which mainly located in the gastrointestinal tract and lungs. The two most common manifestations of this syndrome are diarrhea and facial flushing (Vitale, et al., 2023). While some researchers have uncovered that the release of active substances such as serotonin (5-hydroxytryptamine, 5-HT), histamine, kinins, prostaglandins, and tachykinins was a significant factor in causing CS, the mechanisms behind the occurrence of CS remain unclear (Gade et al., 2020). According to relevant reports, the frequency of CS in NETs patients has increased from 11 to 19% (Halperin et al., 2017). Among the population of patients experiencing CS, those who experience diarrhea and facial flushing can reach as high as 80 to 85% (von der Ohe et al., 1993). Diarrhea is typically the initial symptom in patients with CS, sometimes occurring dozens of times per day. It is often the most distressing symptom experienced by CS patients, significantly reducing their quality of lives and increasing healthcare costs (Kimbrough et al., 2019; Perrier et al., 2023). Therefore, early management and intervention for CS are important.

Both 5-HT and the 5-HT pathway play a crucial role in the pathogenesis of CS (Fanciulli et al., 2020). Most CS patients exhibit alterations in tryptophan metabolism, which typically results in elevated concentrations of 5-HT, thereby activating the 5-HT pathway (Kvols and Reubi, 1993). Consequently, telotristat ethyl, an inhibitor of 5-HT synthesis, has been approved for treating refractory diarrhea in CS, highlighting the significance of the 5-HT pathway in CS (Fanciulli et al., 2020). In CS patients, 5-HT can stimulate intestinal motility and secretion, leading to increased bowel frequency and reduced stool viscosity (Hendrix et al., 1957; von der Ohe et al., 1993). Additionally, other bioactive substances such as prostaglandins also induce intestinal motility and enhance fluid secretion in the gastrointestinal tract, causing diarrhea (Metz et al., 1981). Researchers indicated that substances like prostaglandins, histamine, and substance P can disrupt intestinal secretion and motility, leading to the release of gastrin from enterochromaffin cells in the small intestine. Elevated levels of gastrin can contribute to the cyclic nature of diarrhea (de Celis Ferrari et al., 2018). Moreover, histamine and substance P can cause vasodilation of skin capillaries, resulting in flushing of the skin (Grahame-Smith, 1987). In observations of skin flushing in CS, Schaffalitzky De Muckadell et al. (1986) found increasing concentrations of neurokinin A, neurokinin K, and tachykinin-like peptides. This finding underscored the role of tachykinins in CS. Researchers have reported the presence of substance P, a potent vasodilator, in carcinoid tumor tissue (Ratzenhofer et al., 1981). Evidence also suggested that injecting substance P into healthy individuals can cause transient facial flushing (Schaffalitzky De Muckadell et al., 1986). This finding implied that substance P might be one of the underlying factors contributing to skin flushing in CS. Currently, tryptophan hydroxylase inhibitors and somatostatin analogs are widely used for CS treatment. However, drug resistance and poor tolerability are frequently reported (de Celis Ferrari et al., 2018; Gade et al., 2020). Therefore, there is an urgent need to establish the potential causative relationships in CS, to offer more comprehensive strategies for its treatment.

The intestinal microbiota refers to the community of bacteria, viruses, archaea, fungi, and protozoa that inhabit in the gastrointestinal tract. Numerous studies indicated that tryptophan metabolites play a significant role in regulating gastrointestinal function (Bosi et al., 2020). On the other hand, the intestinal microbiota plays a crucial role in promoting the production of 5-HT. Research has found that metabolites of Clostridium species can upregulate the expression of tryptophan hydroxylase (Tph) gene in enterochromaffin cells, thereby promoting the production of 5-HT. Microbiota-specific metabolites such as short-chain fatty acids, alpha-tocopherol, tyramine, and p-aminobenzoate can stimulate the expression of TPH1 and the release of 5-HT (Yano et al., 2015). In addition, microbes within the human intestines can also produce and degrade histamine (Sanchez-Perez et al., 2022). The gut microbiota and its metabolic product, acetate, can activate the innate immune pathway in intestinal endocrine cells, thereby increasing the secretion of endocrine peptides such as tachykinins (Tumurkhuu et al., 2018). All these findings indicated a potential correlation between gut microbiota and CS, yet currently, there is scarce research focused on this aspect.

Mendelian randomization (MR) study is an analytical method that utilizes genetic variations associated with exposure as instrumental variables to assess potential causal relationships between exposures and outcomes. MR takes advantage of alleles that are randomly segregated during meiotic gamete formation. Since genetic variations precede disease progression and are not influenced by postnatal lifestyle and environmental factors, MR can minimize the impact of confounding factors to a great extent (Sekula et al., 2016).

In this study, a large-scale genome-wide association study (GWAS) dataset was employed to conduct a bidirectional MR analysis, investigating the potential causal relationship between gut microbiota and CS. This approach addresses the existing research gaps in the field and the results will offer novel strategies for the treatment of CS.

## Materials and methods

### Data sources

The gut microbiota abundance data in relation to CS were sourced from the IEU Open GWAS project database,^1^ a database of 246,376,709,462 genetic associations from 42,351 GWAS summary datasets, for querying or download. An exploration was undertaken within the GWAS summary data, utilizing the search query “gut microbiota abundance,” which yielded a total of 211 outcomes. After excluding 15 records that were categorized as “unknown,” 196 relevant results were finally used.

The study of large-scale association analyses identified host factors influencing human gut microbiome composition was curated and analyzed by MiBioGen consortium. This study included genome-wide genotypes and 16S fecal microbiome data from 18,340 individuals (24 cohorts). This study included a total of 211 bacterial taxonomic units, involving 131 genera, 35 families, 20 orders, 16 classes, and 9 phyla (Kurilshikov et al., 2021; MiBioGen consortium, 2023).

The summary data for Genome-Wide Association Study (GWAS) on CS was obtained from the FinnGen biobank analysis round 5. The dataset comprised 16,380,446 SNPs, with 211,123 controls and 161 cases, as reported in the 2021 publication. The participants included individuals of European descent, encompassing both males and females.

### Screening of instrumental variables

In the context of MR study, it is generally required to adhere to three foundational prerequisite assumptions, specifically: (1) the assumption of associativity, (2) the presumption of independence, and (3) the principle of exclusivity (Smith and Ebrahim, 2003).

The assumption of associativity entails that the selected instrumental variables are closely correlated with the exposure of interest, allowing us to confidently employ them as substitutes for the exposure. Typically, we use criteria such as *p* < 1e^−05^, *r*^2^ = 0.001, and Kb = 10,000 as three fundamental thresholds (Sanna et al., 2019). Furthermore, in order to ensure the reliability of these screened instrumental variables, the application of an *F*-test can be employed to eliminate weak instruments. Weak instruments are commonly defined by an F-statistic value of less than 10. The formula to calculate *F*-statistic value is as follow:

$F={\left(\frac{\mathrm{Beta}}{SE}\right)}^{2}$ (Casas et al., 2006).

Among them, Beta refers to the effect size of the SNP on the exposure, and SE (Standard error) refers to the standard error of Beta. The assumption of independence in MR refers to the genetic variants (genotypes or genetic variations) being unrelated to other factors that could potentially affect the outcomes when they are randomly allocated. The assumption of independence necessitates that the distribution of genotypes among participants is random and not influenced by other possible confounding factors.

The assumption of exclusivity refers to the genetic variants (genotypes or genetic variations) being allocated among participants in a mutually exclusive manner, with each participant being assigned to a specific genotype only. This assumption ensures that genotypes do not overlap or coexist among participants, thereby allowing the association between genotypes and exposure to be accurately interpreted and assessed.

To ensure the enforcement of the aforementioned-assumptions, we subjected the selected instrumental variables (IVs) to tests for horizontal pleiotropy and heterogeneity. For the assessment of pleiotropy, we utilized the Egger intercept and MR Presso test. Heterogeneity assessment was carried out using the heterogeneity function of the TwoSampleMR package in R language 4.3.1. Furthermore, we conducted Steiger test to ascertain the exclusion of SNPs with reverse causal relationships. Leave-one-out sensitivity analysis was employed to evaluate the stability of each SNP’s influence on the outcome.

### Statistical analysis

IVW is used as the primary method to assess the causal relationship between gut microbiota abundance and CS. The strength of IVW lies in its ability to provide a more robust outcome; if a SNP in the instrumental variables is invalid, it can introduce bias to the results (Burgess et al., 2016). Additionally, we employed three alternative methods: MR-Egger regression, weighted median model, and weighted mode. MR-Egger regression is a technique that refines the IVW method. It takes into consideration the intercept term in the regression model to detect and correct for pleiotropy effects. It relies on the assumptions of the InSIDE (Instrument Strength Independent of Direct Effect) and NOME (No Measurement Error) principles (Bowden et al., 2015). Weighted median model and weighted mode share similarities in using the reciprocal of outcome variance as weights. The difference lies in their methods of aggregation. The weighted median model employs a weighted median approach, while the weighted mode employs a weighted mode approach.

## Results

### Characteristics of SNPs

According to the filtering criteria of *p* < 1e^−05^, *r*^2^ = 0.001, and Kb = 10,000, a total of 224, 434, 512, 486, 498, 280, and 125 SNPs were, respectively, obtained from the class, family, genus1, genus2, genus3, order, and phylum of the gut microbiota. Additionally, from the outcome “Carcinoid symptom,” 179, 339, 413, 395, 391, 217, and 103 SNPs were extracted for analysis. The *F*-values of all instrumental variables were greater than 10, indicating no weak instrumental variables in this study. The detail of the characteristics of SNPs were shown in Supplementary Table S1.

### Mendelian randomization analysis

The IVW results indicated that a total of 8 types of gut microbiota are associated with CS. As the IVW results of Gut microbiota abundance (class Coriobacteriia id.809), Gut microbiota abundance (family Coriobacteriaceae id.811), and Gut microbiota abundance (order Coriobacteriales id.810) were consistent, for a more precise outcome, we retained only the lowest taxonomic level, Gut microbiota abundance (family Coriobacteriaceae id.811), for presentation. This yielded a total of 6 gut microbiota types that are correlated with CS.

**Figure 1:**
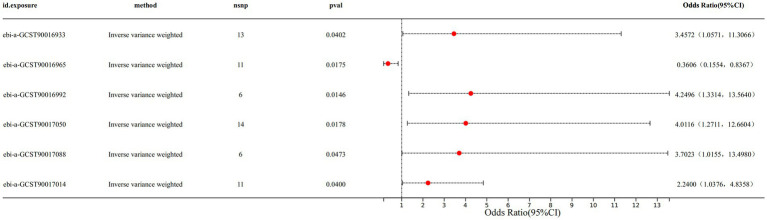
The IVW results of 6 gut microbiota against CS.

Specifically, we found that genus Anaerofilum (IVW odds ratio [OR] = 0.3606, 95% confidence interval [CI]: 0.1554–0.8367, *p* = 0.0175) had a protective effect on CS. While family Coriobacteriaceae (IVW OR = 3.4572, 95%CI: 1.0571–11.3066, *p* = 0.0402), genus Enterorhabdus (IVW OR = 4.2496, 95%CI: 1.3314–13.5640, *p* value: 0.0146), genus Ruminiclostridium6 (IVW OR: 4.0116, 95%CI: 1.2711–12.6604, p value: 0.0178), genus Veillonella (IVW OR: 3.7023, 95%CI: 1.0155–13.4980, p value: 0.0473) and genus Holdemanella (IVW OR: 2.2400, 95%CI: 1.0376–4.8358, p value: 0.0400) demonstrated a detrimental effect on CS (Figure 1 and Supplementary Table S2). The forest plot displayed the odds ratio (OR) and 95% confidence interval for each SNP, followed by the aggregation of all SNPs using IVW and MR Egger (Supplementary Figure S1). The scatter plot illustrated the effect distribution of all SNPs, demonstrating trends for four different MR analysis methods (Supplementary Figure S2).

The heterogeneity test indicated that there was no heterogeneity in the above results for the SNPs. The funnel plot showed that all SNPs are distributed evenly on both sides of a straight line, which confirms this observation (Supplementary Table S3 and Supplementary Figure S3). The Egger intercept and MR presso test indicated the absence of pleiotropy in the above results for the SNPs, demonstrating the reliability of this study (Supplementary Tables S4, S5). The Leave-one-out sensitivity analysis was conducted by excluding individual SNPs to assess the overall effect change, and no single SNP was found to have a significant impact on the outcome (Supplementary Figure S4). Lastly, we did not observe a reverse causal relationship between CS and the afore-mentioned gut microbiota.

## Discussion

This study represented the inaugural attempt to investigate the causal relationship between gut microbiota and carcinoid syndrome (CS) utilizing a bidirectional MR analysis. The findings indicated the presence of causal associations between 6 specific gut microbiota taxa and CS, while no reverse causal relationship exists between CS and gut microbiota. At present, there was no prior research delved into the relationship between gut microbiota and CS.

CS is a collection of clinical symptoms caused by excessive secretion of mediators such as serotonin (5-HT), substance P, histamine, and prostaglandins (Yano et al., 2015). Research has shown a close correlation between gut microbiota and certain bioactive substances, but the relationship between gut microbiota and CS remains unclear. A prior study indicated that microbial metabolites in the gut, such as short-chain fatty acids, α-tocopherol, tyramine, and p-aminobenzoate, can stimulate the release of 5-HT (Yano et al., 2015). Moreover, the human gut microbiota can both produce and degrade histamine (Sanchez-Perez et al., 2022). These studies highlighted a potential correlation between gut microbiota and CS; however, further evidence is needed to confirm this association. This study employed MR analysis to reveal causal relationships between 6 specific gut microbiota taxa and CS, addressing the gaps in existing research and contributing to the refinement of therapeutic approaches for CS.

Our study revealed the causal relationships between a total of 6 specific gut microbiota taxa and CS. Among them, genus Anaerofilum was the only protective bacterial group identified in CS. Research indicated that the expression of functional genes in genus Anaerofilum effectively promoted the tryptophan-indole metabolic pathway in the intestines (Sun M. et al., 2020; Sun X. Z. et al., 2020). *In vitro* experiments have demonstrated that adding a certain concentration of indole induces the expression of tight junction proteins in intestinal epithelial cells, thereby restoring intestinal barrier function (Sun M. et al., 2020; Sun X. Z. et al., 2020). Intestinal barrier function is primarily provided by the tight junctions of adjacent epithelial cells (Camilleri et al., 2017), and disruption of tight junction function has been observed to lead to diarrhea in animal models (Halliez et al., 2016). Additionally, aside from the indole pathway, tryptophan also participates in the kynurenine pathway and the serotonin (5-HT) pathway. Prior studies showed that nearly all CS patients experience abnormal tryptophan metabolism, leading to a significant increase in blood 5-HT concentrations. 5-HT and its metabolites are believed to play a crucial role in the development of typical symptoms in CS patients (Fanciulli et al., 2020). These symptoms include diarrhea (von der Ohe et al., 1993; Boutzios and Kaltsas, 2015), intestinal obstruction, and others (Connolly and Pellikka, 2006; Hannah-Shmouni et al., 2016). Therefore, the protective effect of genus Anaerofilum in CS may primarily exist in two aspects: On the one hand, genus Anaerofilum directly reduces the occurrence of diarrhea by promoting the tryptophan-indole metabolic pathway to repair the intestinal barrier; On the other hand, the active tryptophan-indole pathway effectively inhibits the tryptophan-5-HT pathway, reducing intestinal motility and secretory reflex, thereby indirectly improving diarrhea symptoms.

In addition to the genus Anaerofilum, we identified 5 other gut microbial populations as risk factors for CS. In a murine model, it was discovered that genus Enterorhabdus showed a positive correlation with tryptophan levels and inhibited the indoleamine pathway of tryptophan metabolism (Deng et al., 2021). As mentioned above, inhibiting the indoleamine pathway of tryptophan metabolism can indirectly increase the concentration of 5-HT. Additionally, the genus Enterorhabdus is also associated with intestinal barrier function. A study indicated that the genus Enterorhabdus can increase the production of lyso-phosphatidylcholine, thereby promoting the release of pro-inflammatory cytokines and damaging the intestinal epithelial barrier of murine (Tang et al., 2021).

The family Coriobacteriaceae was a significant risk factor for inducing CS. Research has shown that Coriobacteriaceae UCG-002 can produce cytotoxic compounds such as phenol and *p*-cresol, consequently altering epithelial permeability and reducing epithelial barrier function (Saito et al., 2018; Yu et al., 2023). Simultaneously, Tian et al. (2023) also observed an increased relative abundance of Coriobacteriaceae UCG-002 in cases of intestinal damage, and a positive correlation between Coriobacteriaceae UCG-002 and the inflammatory cytokine TNF-α. TNF-α, a type of tumor necrosis factor (TNF), inhibits the Wnt/β-catenin pathway, thereby compromising the stability of intestinal epithelium (Wu et al., 2020). Therefore, the family Coriobacteriaceae may potentially exacerbate certain symptoms in patients with carcinoid syndrome by inducing the production of various harmful mediators that damage the epithelial barrier function.

In a MR study exploring the relationship between gut microbiota and asthma, genus Ruminiclostridium 6 was found to be associated with the incidence of moderate to severe asthma (Li et al., 2023). Bronchial asthma is a heterogeneous disease characterized by chronic inflammation of the airways (Kaczynska et al., 2021). Certain cells such as eosinophils, neutrophils, and endogenous inflammatory mediators like leukotrienes and histamine participate in the inflammatory processes in the airways (Barnes, 2008). Additionally, many regulatory peptides such as kinins are shown to be involved in the regulation of asthma-related inflammation and airway hyperresponsiveness (Kaczynska et al., 2018; Pavon-Romero et al., 2021). Interestingly, about 20% of CS patients also experience bronchoconstriction mediated by kinins and bradykinins (Cunningham et al., 2008).

In addition to the previously mentioned 5-HT and bradykinin, histamine also plays a significant role in CS. Researchers suggested that histamine might be a potential mediator for the facial flushing and bronchospasm symptoms observed in patients with colorectal CS (de Celis Ferrari, et al., 2018). The pathogenic bacterium genus Veillonella was confirmed to have a strong ability to induce mast cells to release histamine (Nygren and Dahlen, 1981). Furthermore, in fecal samples from individuals with higher asthma frequencies, genus Veillonella was found to be enriched, and metabolic profiling indicated the importance of histidine metabolism in the asthma process, which leads to the formation of histamine upon histidine decarboxylation (Lee-Sarwar et al., 2022). Therefore, genus Veillonella might exacerbate the development of CS by inducing histamine production.

The genus Holdemanella is also identified as a risk factor for CS. However, at present, there are no reports linking this genus to endocrine mediators associated with CS. Hu et al. (2020) discovered that the expression of tight junction proteins in the ileum was significantly increased, and the relative abundance of genus Holdemanella in the gut microbiota was reduced in a weaned piglet model supplemented with catechin. Therefore, we speculated that genus Holdemanella might exacerbate diarrhea symptoms in patients with CS by potentially affecting intestinal mucosal barrier function.

## Conclusion

In conclusion, this study utilized a bidirectional mendelian randomization analysis and identified 6 gut microbial populations that are causally associated with carcinoid syndrome. This research represented the first instance of uncovering a causal link between gut microbiota and CS, offering a novel strategy for its treatment. Further validation through additional randomized controlled trails are warranted in the future to solidify these findings.

## Data availability statement

The original contributions presented in the study are included in the article/Supplementary material, further inquiries can be directed to the corresponding author.

## Author contributions

ZZ: Conceptualization, Methodology, Writing – original draft. DL: Writing – original draft. FX: Writing – original draft. GM: Investigation, Writing – original draft. HZ: Writing – review & editing.

## Footnotes

^1^https://gwas.mrcieu.ac.uk/
