# Supplementary figures and images for: The cause-and-effect relationship between gut microbiota abundance and carcinoid syndrome: a bidirectional Mendelian randomization study
Source: Front Microbiol. 2023 Dec 22;14:1291699. doi: 10.3389/fmicb.2023.1291699 (PMC10766758; doi:10.3389/fmicb.2023.1291699)

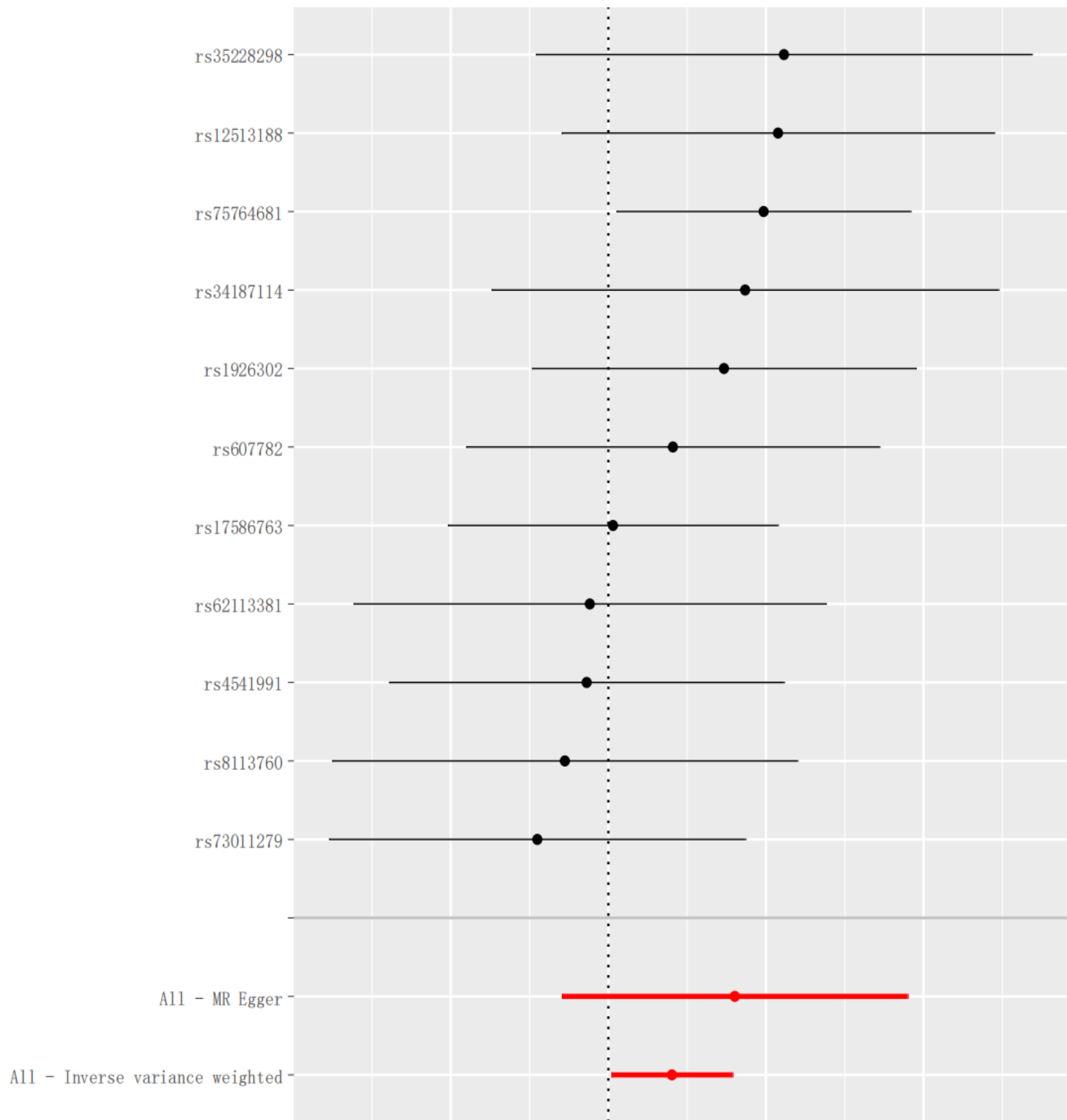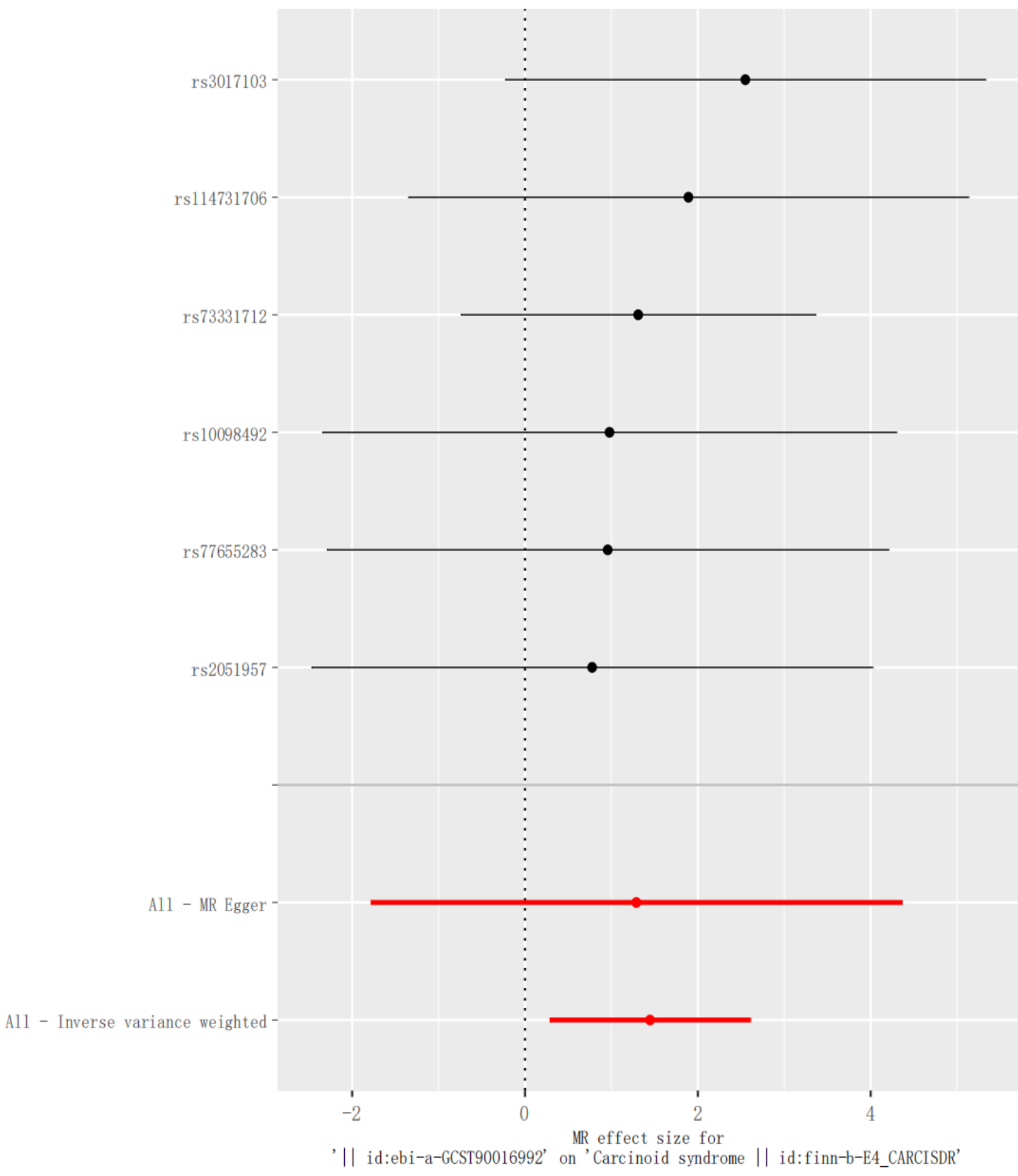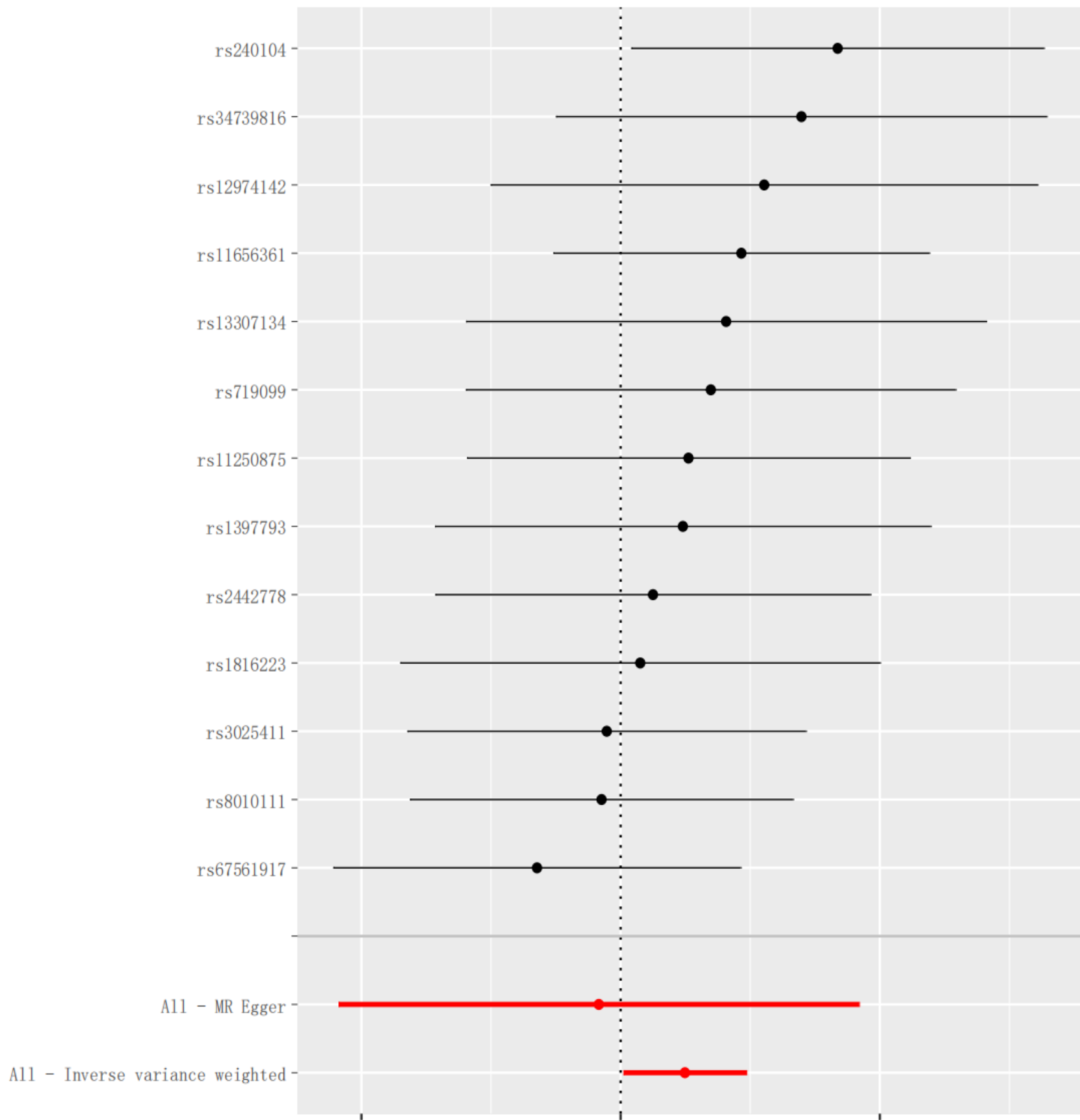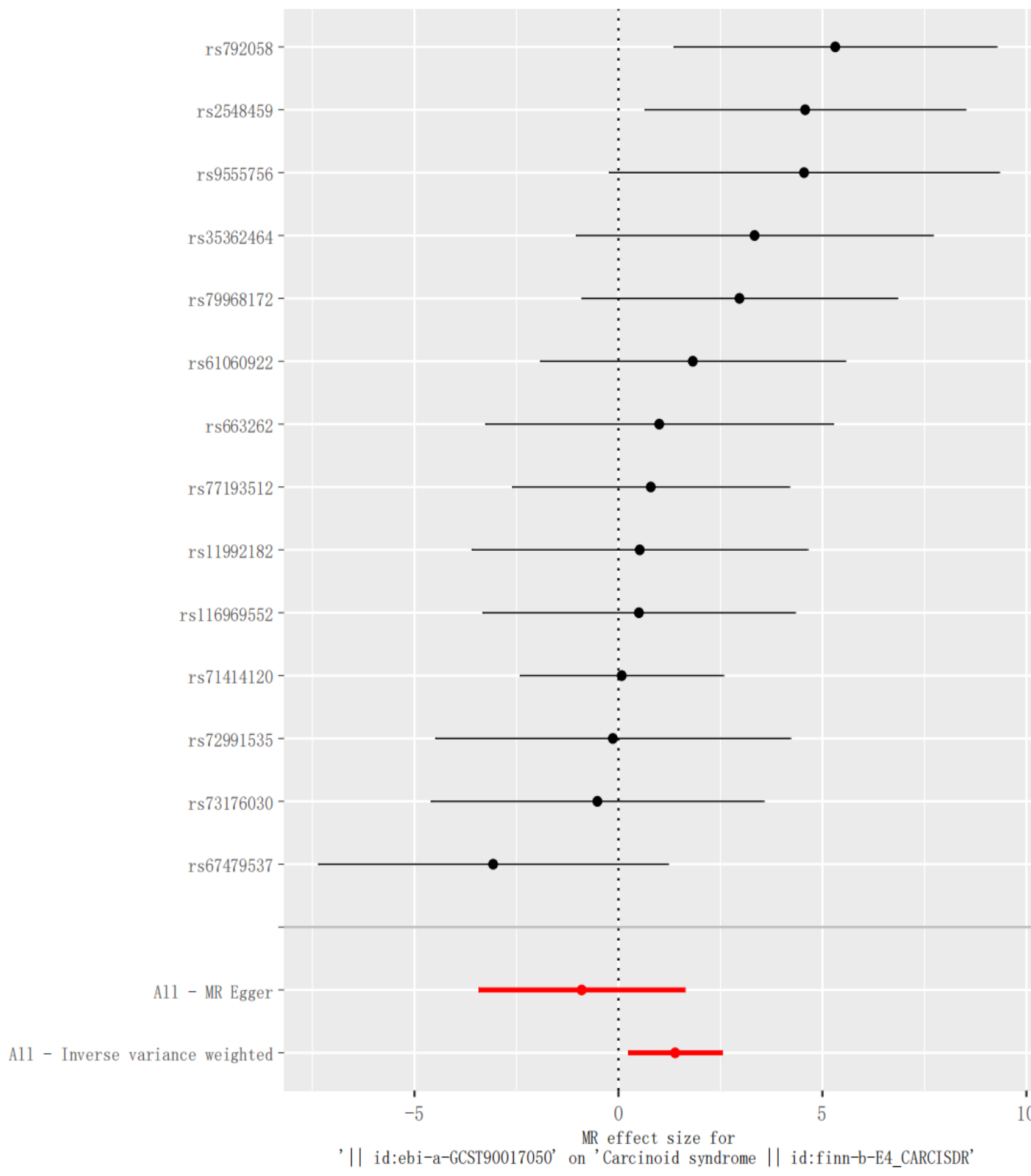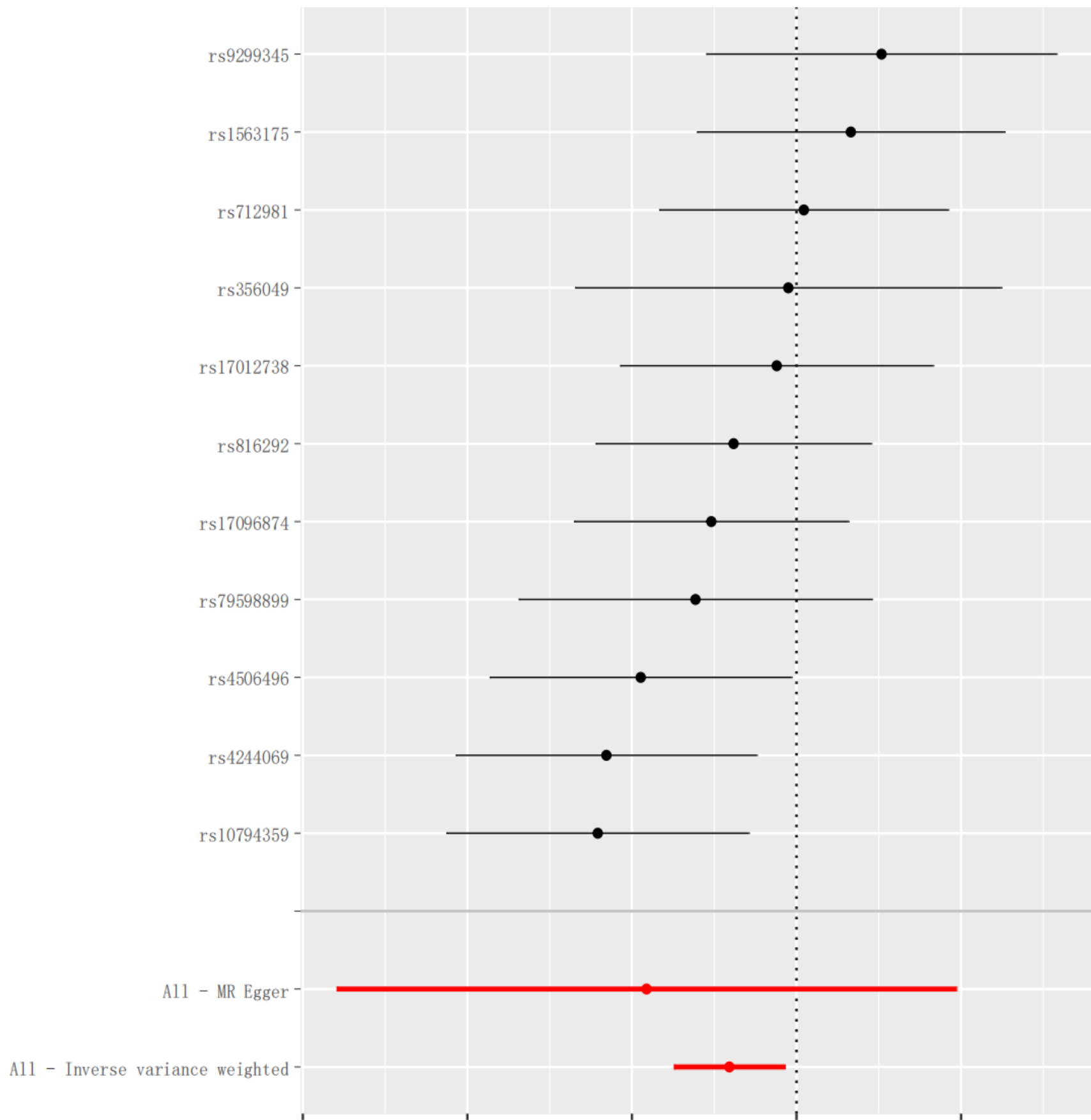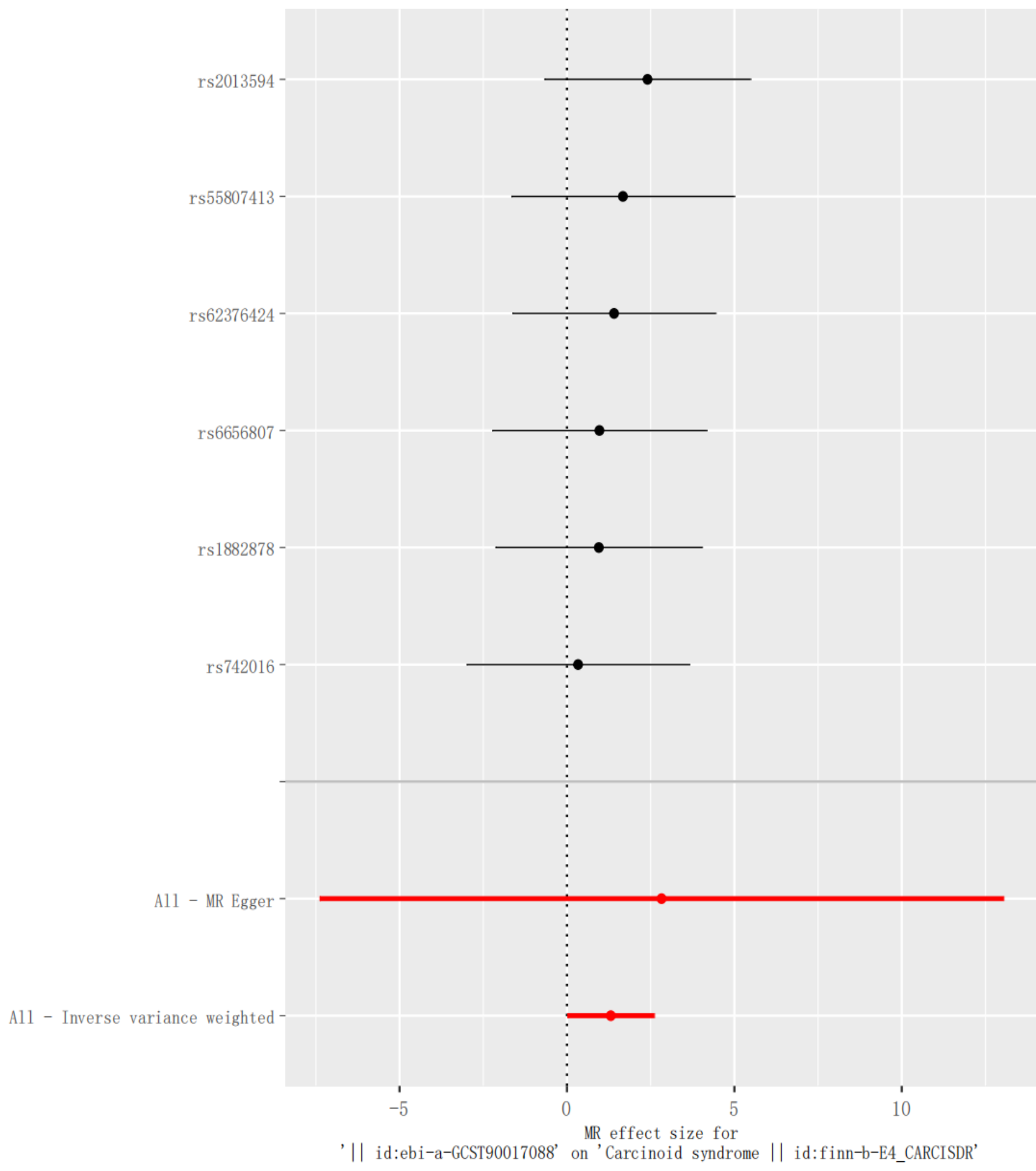

Supplement: Supplementary file 1 [file Data_Sheet_1.PDF]

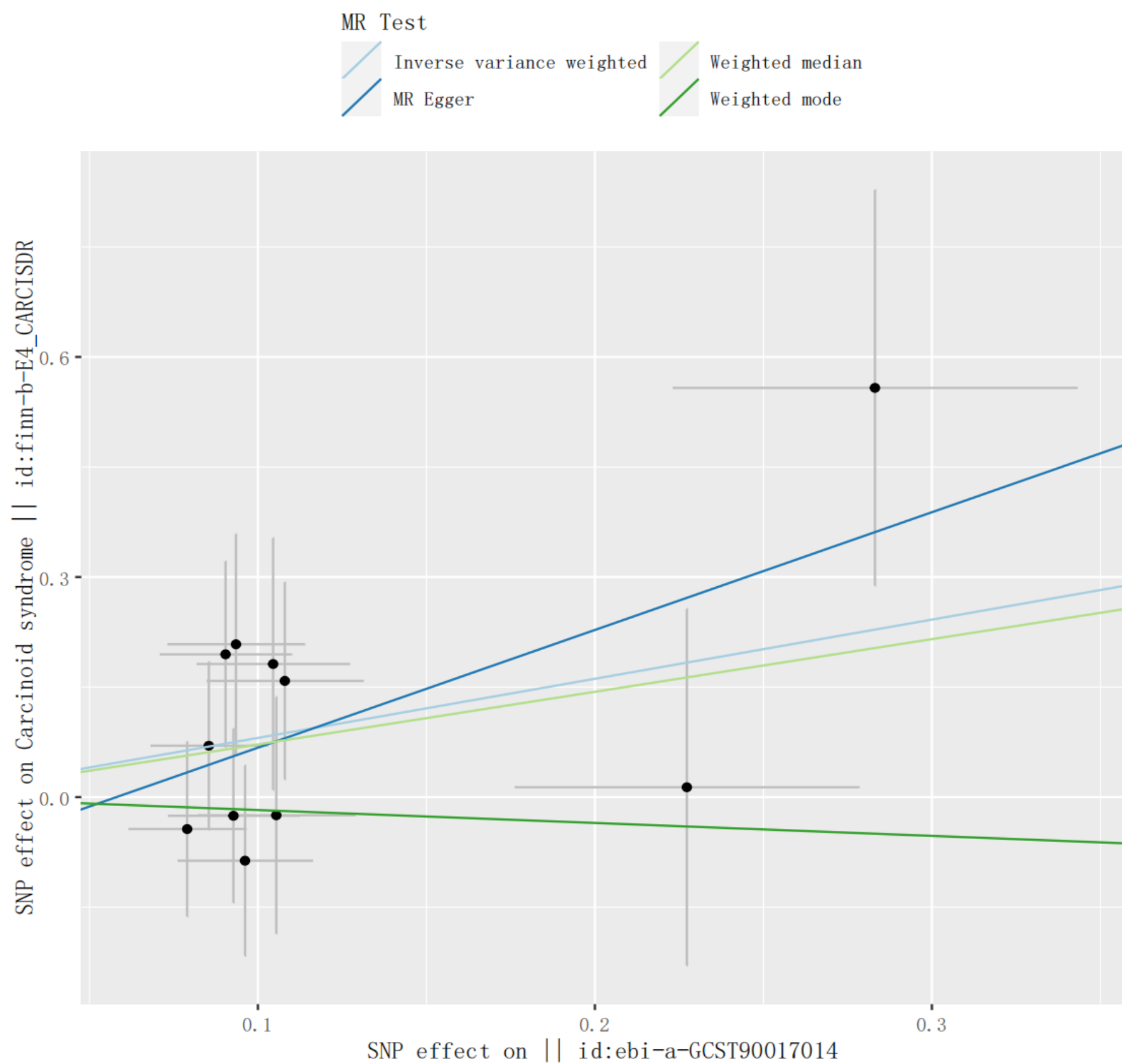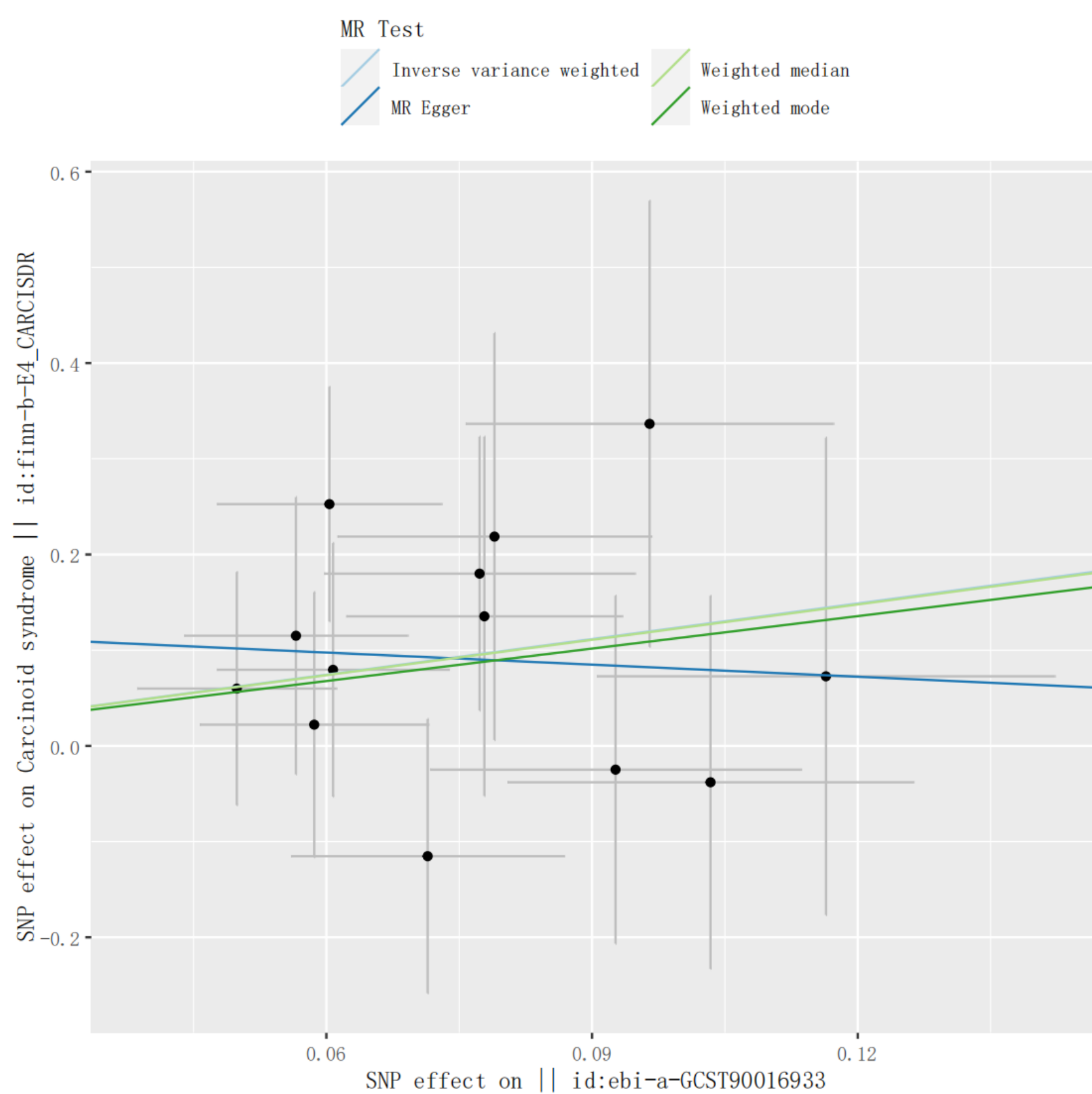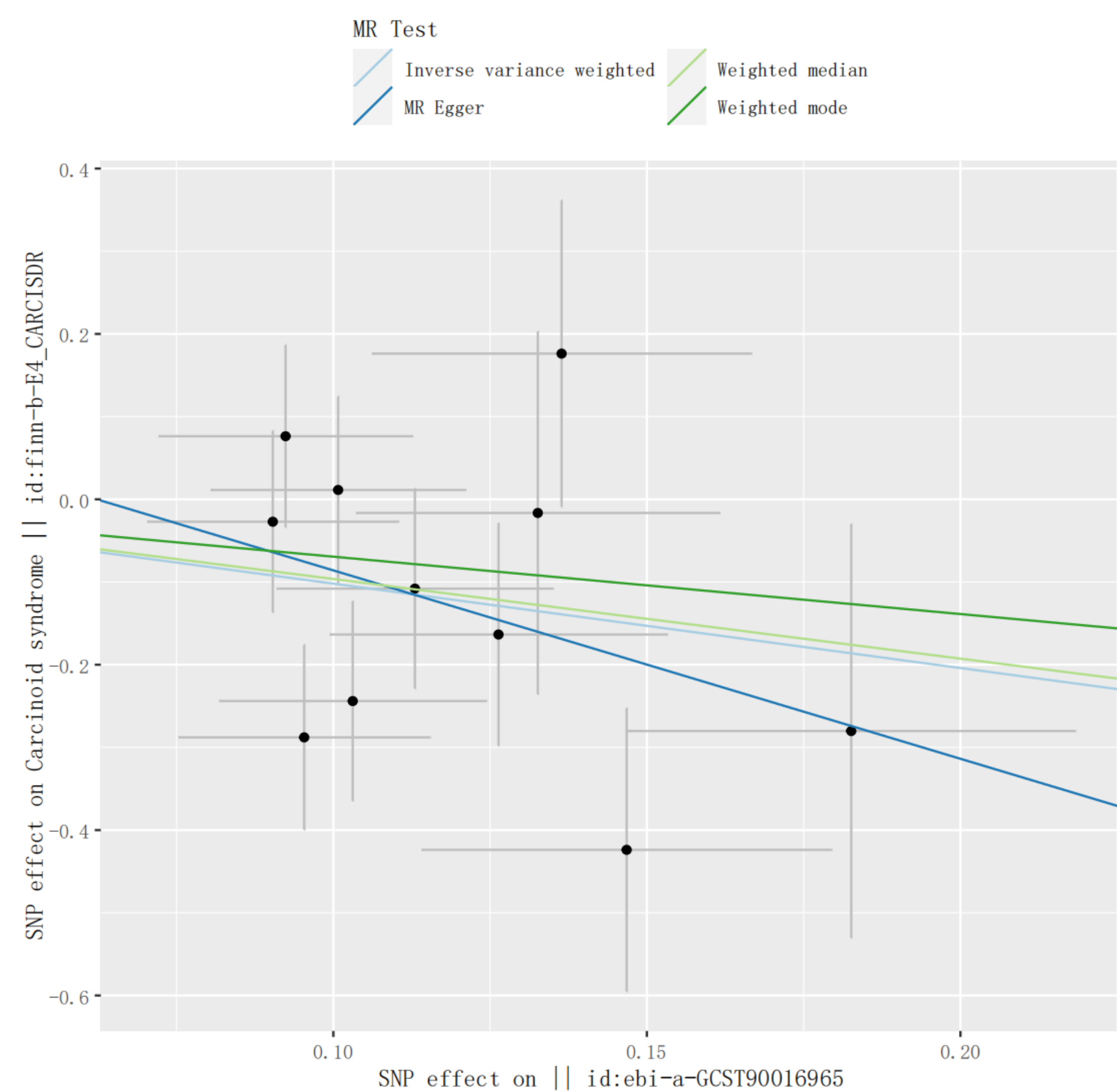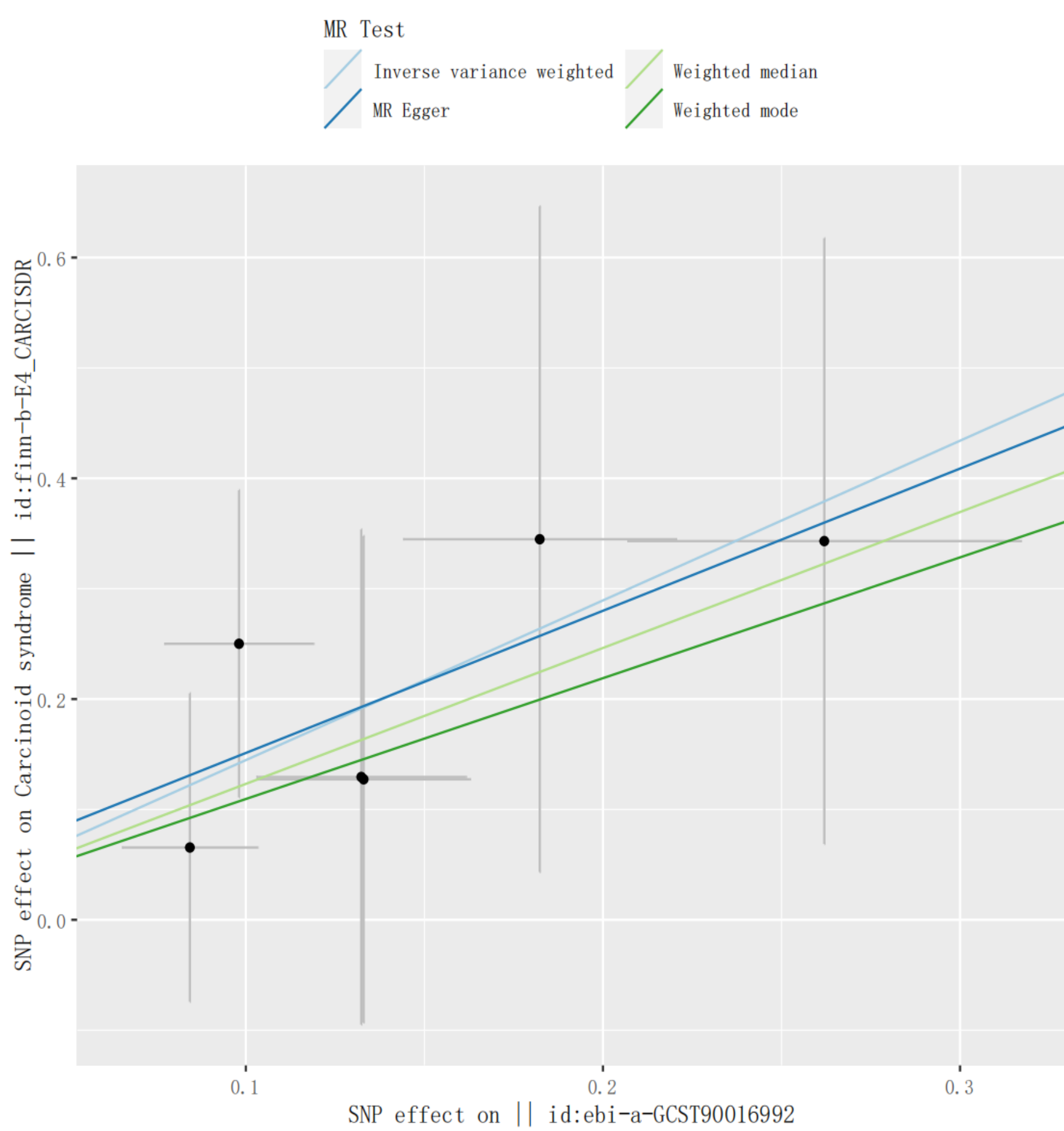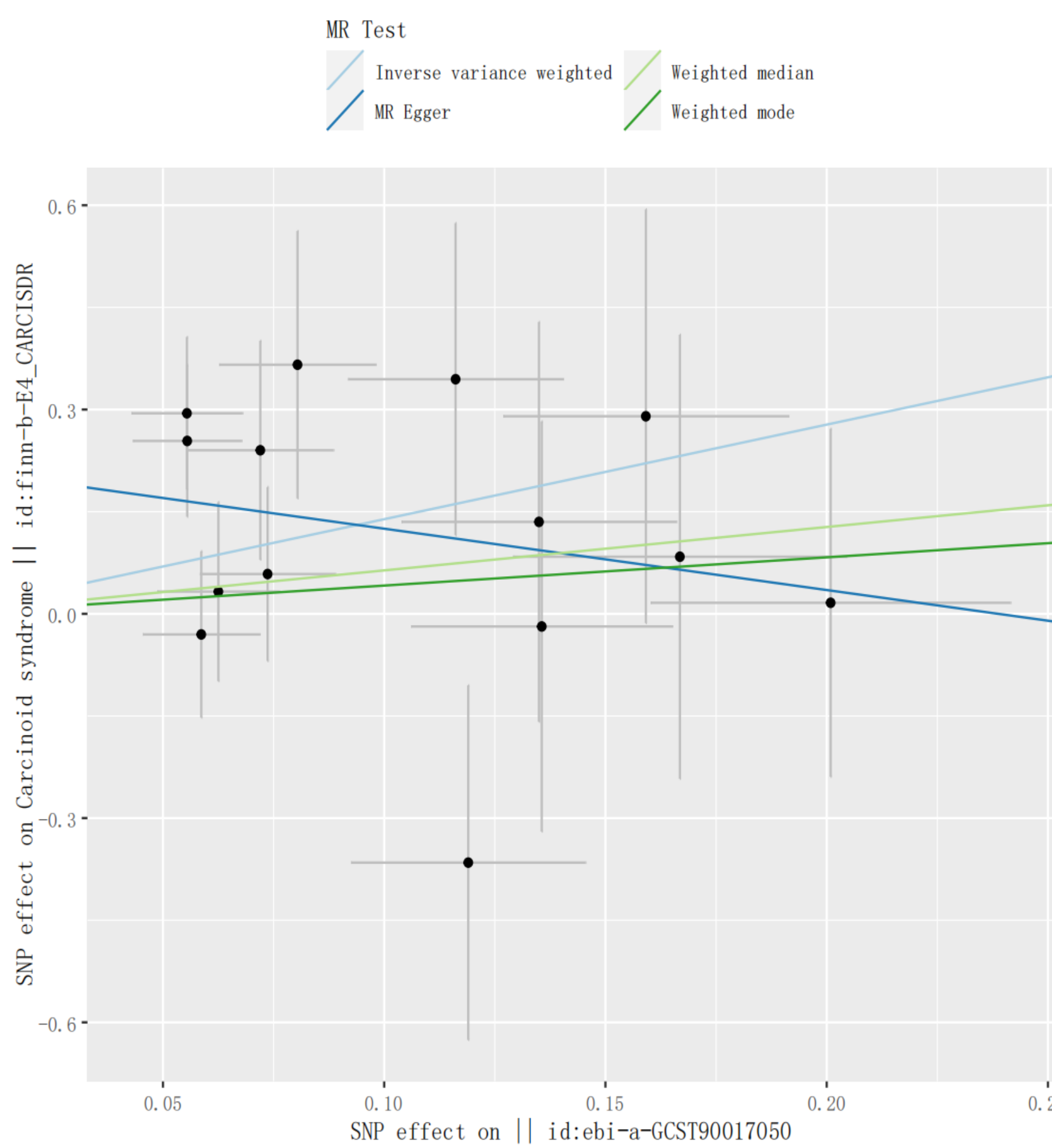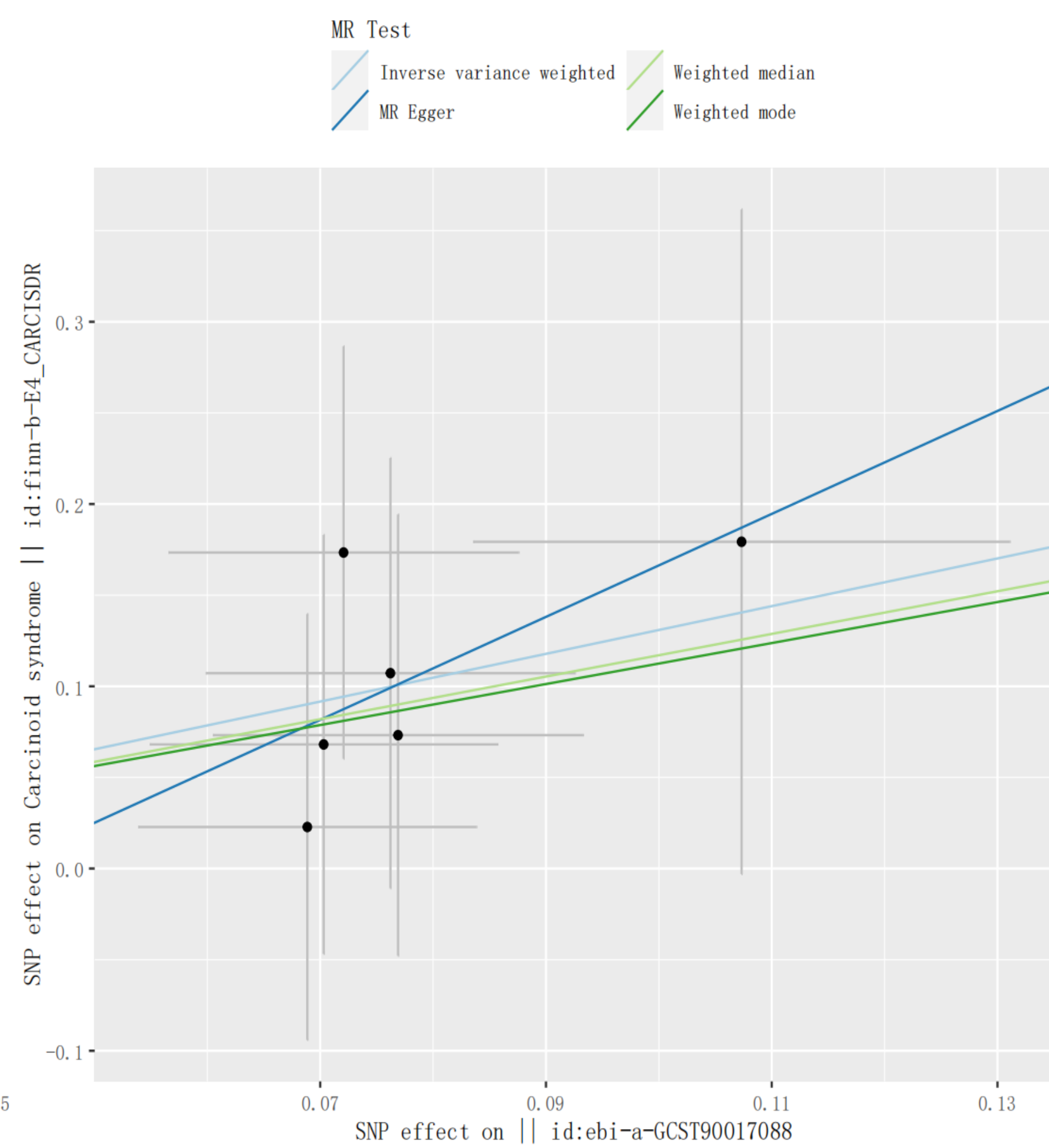

Supplement: Supplementary file 2 [file Data_Sheet_2.PDF]
